# iPSC‐derived human cortical organoids display profound alterations of cellular homeostasis following SARS‐CoV‐2 infection and Spike protein exposure

**DOI:** 10.1096/fj.202401604RRR

**Published:** 2025-02-14

**Authors:** Gioia Cappelletti, Lorenzo Brambilla, Sergio Strizzi, Fiona Limanaqi, Valentina Melzi, Mafalda Rizzuti, Monica Nizzardo, Irma Saulle, Daria Trabattoni, Stefania Corti, Mario Clerici, Mara Biasin

**Affiliations:** ^1^ Department of Biomedical and Clinical Sciences University of Milan Milan Italy; ^2^ Neurology Unit Foundation IRCCS Ca’ Granda Ospedale Maggiore Policlinico Milan Italy; ^3^ Department of Pathophysiology and Transplantation University of Milan Milan Italy; ^4^ Department of Pathophysiology and Transplantation (DEPT), Dino Ferrari Centre, Neuroscience Section University of Milan Milan Italy; ^5^ Neuromuscular and Rare Diseases Unit, Department of Neuroscience Fondazione IRCCS Ca' Granda Ospedale Maggiore Policlinico Milan Italy; ^6^ Don C. Gnocchi Foundation Istituto di Ricovero e Cura a Carattere Scientifico (IRCCS) Foundation Milan Italy

**Keywords:** COVID‐19, iPSC‐derived human cortical organoids, long‐COVID, neuroinflammation, SARS‐CoV‐2 infection, Spike protein

## Abstract

COVID‐19 commonly leads to respiratory issues, yet numerous patients also exhibit a diverse range of neurological conditions, suggesting a detrimental impact of SARS‐CoV‐2 or the viral Spike protein on the central nervous system. Nonetheless, the molecular pathway behind neurological pathology and the presumed neurotropism of SARS‐CoV‐2 remains largely unexplored. We generated human cortical organoids (HCOs) derived from human induced pluripotent stem cells (hiPSC) to assess: (1) the expression of SARS‐CoV‐2 main entry factors; (2) their vulnerability to SARS‐CoV‐2 infection; and (3) the impact of SARS‐CoV‐2 infection and exposure to the Spike protein on their transcriptome. Results proved that (1) HCOs express the main SARS‐CoV‐2 receptors and co‐receptors; (2) HCOs may be productively infected by SARS‐CoV‐2; (3) the viral particles released by SARS‐CoV‐2‐infected HCOs are able to re‐infect another cellular line; and (4) the infection resulted in the activation of apoptotic and stress pathways, along with inflammatory processes. Notably, these effects were recapitulated when HCOs were exposed to the Spike protein alone. The data obtained demonstrate that SARS‐CoV‐2 likely infects HCOs probably through the binding of ACE2, CD147, and NRP1 entry factors. Furthermore, exposure to the Spike protein alone proved sufficient to disrupt their homeostasis and induce neurotoxic effects, potentially contributing to the onset of long‐COVID symptoms.

## Graphical abstract

Graphical representation of the study workflow. iPSC‐derived human cortical organoids (HCOs) at Day 40, were in vitro infected by SARS‐CoV‐2 or exposed to the Spike protein and viral infection/replication was assessed by a multidisciplinary approach using RT‐qPCR, TCID50, and immunofluorescence. SARS‐CoV‐2 host entry factor expression was assessed on HCOs by digital‐PCR and immunofluorescence. The effect of viral infection on HCOs homeostasis was determined by analyzing the alteration of their transcriptome, while the stress pathway was investigated by immunofluorescence assay.

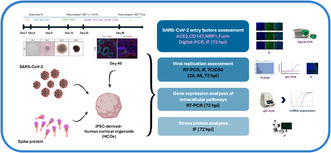

## 1 INTRODUCTION

Neurological complications have been linked to COVID‐19, even in individuals with mild symptoms.^1^, ^2^, ^3^ Indeed, up to 65% of COVID‐19 patients report diverse neural symptoms, potentially contributing to the “long‐COVID syndrome.”^4^, ^5^, ^6^, ^7^, ^8^ These complex neurological manifestations impact both the central and the peripheral nervous systems,^9^, ^10^, ^11^, ^12^, ^13^, ^14^, ^15^, ^16^, ^17^, ^18^, ^19^, ^20^, ^21^, ^22^, ^23^, ^24^ suggesting a potential acceleration of neurodegeneration and an increased risk of neurodegenerative diseases, in line with studies on other viral infections.^25^, ^26^, ^27^, ^28^, ^29^, ^30^, ^31^, ^32^ However, it is still debated whether COVID‐19‐associated neurological manifestations result from direct neuronal infection, neural damage induced by viral proteins, immune‐mediated syndromes subsequent to the infection, or consequences of systemic illness.^1^, ^33^, ^34^

Evidence suggests that the viral proteins may act like pathogen‐associated molecular patterns (PAMPs), and cause neuroinflammatory conditions even without direct central nervous system (CNS) infection. In particular, the SARS‐CoV‐2 spike (S) protein, can stimulate brain endothelial cells to respond in a pro‐inflammatory manner, potentially altering the functions and properties of the blood–brain barrier (BBB).^35^ Additionally, SARS‐CoV‐2 S protein may induce phenotypic modifications in hematopoietic cells^36^ and stimulate human microglia's NLRP3 inflammasome via NF‐κB and ACE2.^37^ Furthermore, studies using autopsies from COVID‐19 patients^38^, ^39^, ^40^, ^41^, ^42^, ^43^, ^44^, ^45^, ^46^, ^47^, ^48^, ^49^ and animal models^38^, ^47^, ^48^ showed evident microglial activation and significant neuroinflammation in the brain stem,^37^ possibly exacerbating neurological and neuropsychiatric symptoms. These results indicate that COVID‐19‐related neuropathological effects may arise from the direct impact of the virus on the CNS and/or may be triggered via viral protein‐mediated inflammation. In this context, revealing the effects of the virus and viral proteins on various types of brain cells represents a priority, especially considering the likelihood of SARS‐CoV‐2 neuroinvasiveness.^50^

Human brain tissue is nearly impossible to study, mainly in patients infected with contagious pathogens,^51^ and 2D mono‐cell cultures are not suitable for demonstrating the effects of viruses on multicellular networks or tissue integrity. Thus, induced pluripotent stem cells (iPSCs)‐derived human brain organoids provide a viable and safe alternative and represent a suitable platform to explore the direct effects of SARS‐CoV‐2 on the CNS, as recently reviewed by Ostermann et al.^52^ Indeed, this 3D in vitro model has proven to be highly effective to explore human brain development and different neurological conditions^53^, ^54^, ^55^, ^56^, ^57^, ^58^, ^59^ as well as to investigate the susceptibility to SARS‐CoV‐2 infection.^60^, ^61^, ^62^, ^63^, ^64^, ^65^, ^66^, ^67^, ^68^, ^69^, ^70^, ^71^

However, the molecular cascade triggered by SARS‐CoV‐2 infection and/or S exposure, along with the SARS‐CoV‐2 possible neurotropism in neuronal cells, have been only partially unveiled.

To tackle this issue, by adopting an experimental approach recently used to investigate motor neuron susceptibility to SARS‐CoV‐2,^10^ we assessed iPSC‐derived human cortical organoids (HCOs) for (i) the presence of key human receptors and co‐receptors for SARS‐CoV‐2 entry; (ii) their susceptibility to SARS‐CoV‐2 infection; (iii) the effects of SARS‐CoV‐2 infection and S protein exposure on their transcriptome; and (iv) protein expression.

## 2 MATERIALS AND METHODS

### 2.1 Cell lines, virus, and reagents

Vero E6 cells (ATCC, VA, USA) were grown in DMEM (Dulbecco's Modified Eagle's Medium) (Euroclone, Milan, Italy) with 10% FBS, 4 mM l‐glutamine, 100 μg/mL streptomycin, and 100 U/mL penicillin at 37°C and 5% CO_2_. Regular mycoplasma contamination checks were performed using PCR, and experiments were carried out with cells from passages 15 to 25. The Delta (B.1.617.2) SARS‐CoV‐2 lineage (kindly provided by the Clinical Microbiology, Virology and Bioemergency diagnosis Unit—ASST Fatebenefratelli Milan, Italy) was grown into VeroE6 cells to create a viral stock. The concentration of viral particles was calculated as 50 percentage tissue culture infectious dose (TCID_50_), as previously described.^72^ SARS‐CoV‐2 recombinant S protein was acquired from BEI Resources NIAID, NIH (Cat. N. NR52397; www.beiresources.org).

### 2.2 iPSCs generation and HCOs differentiation

Induced pluripotent stem cells (iPSCs) were generated from peripheral blood mononuclear cells (PBMCs) obtained from a total of four healthy subjects (Table 1), through a non‐integrating reprogramming protocol via a modified protocol of Sendai Virus to deliver the four reprogramming factors: Oct4, Sox2, Klf4, and c‐Myc (CytoTune®‐iPSC 2.0 Sendai Reprogramming kit, Thermo Fisher Scientific). iPSCs were cultured in Cultrex‐coated (1% for 1 h at 37°C) 6‐well plates and grown in complete Essential 8™ Medium (E8, Thermo Fisher Scientific). Before proceeding with the HCO generation, iPSCs were checked for chromosomal abnormalities by karyotyping. Furthermore, the vector‐free status of iPSCs clones was assessed by performing an RT‐PCR for the detection of the SeV genome and transgenes.

**TABLE 1:** Sex, age, and number of independent replicates performed for each assay with iPS‐HCOs lines obtained from four healthy donors are reported.

	Sex	Age	Real‐time PCR for N1 and N2 viral genes	TCID50 on VERO E6 cells	Gene expression analysis by real‐time PCR	Gene expression by digital	IF
S1	M	69	*n* = 3	*n* = 3	*n* = 3	*n* = 3	*n* = 3
S2	F	64	*n* = 3	*n* = 3	*n* = 3	*n* = 3	*n* = 3
S3	M	65	*n* = 3	*n* = 3	*n* = 3	*n* = 3	*n* = 3
S4	F	33	*n* = 3	*n* = 3	*n* = 3	–	*n* = 3

Abbreviation: S, subject.

iPSCs were differentiated into HCOs based on the approach that Miura et al.^73^ detailed. Upon reaching 80%–90% confluence, the cells were seeded at a density of 10^4^ cells per well in a 96‐well plate, using complete E8TM Medium supplemented with 10 μM Rock inhibitor. Accutase was used to detach the cells (5 min, 37°C). iPSCs were grown in 96 well plates to form embryoid bodies (EBs) until DIV12. From DIV0 to DIV 6, E8™ medium was enriched with 2.5 μM dorsomorphin (DM) and 10 μM SB‐431542 with changes made every other day. At DIV6, the neural induction medium was replaced with neural differentiation one, consisting of Neurobasal medium, 2% B27 supplement, 1% Glutamax, and 1% penicillin–streptomycin, supplemented with 20 ng/mL epidermal growth factor (EGF) and 20 ng/mL basic fibroblast growth factor (bFGF). Up until DIV22, the medium was switched every 2 days. At DIV12, EBs were transferred in 6 well plates. From DIV22 to DIV40, EGF and bFGF were replaced with 20 ng/mL brain‐derived neurotrophic factor (BDNF), 20 ng/mL neurotrophin‐3 (NT3), 200 μM ascorbic acid (AA), 50 μM dibutyryl‐cAMP sodium salt, and 10 μM docosahexaenoic acid (DHA). At DIV40, cortical organoids were used for the infection assay.

### 2.3 HCO in vitro SARS‐CoV‐2 infection assay

In vitro HCOs infection was performed with SARS‐CoV‐2 at 0.001 MOI (multiplicity of infection). The MOI was calculated considering a value of 3 × 10^5^ cells per single HCO. Following overnight incubation, HCOs were rinsed with PBS and filled with a complete neural differentiation medium. For the assessment of SARS‐CoV‐2 replication and the execution of a re‐infection test on VeroE6 cells in quadruplicate, supernatants were harvested at four different time points. The first point was set at an early stage of infection, whereas the last one was set in a late stage to appreciate viral infection‐kinetics over time. Thus, time point −0 (T0), −1 (T1), −2 (T2), and −3 (T3) have been scheduled at 6‐, 24‐, 48‐, and 72 h post‐infection (hpi), respectively. At 72 hpi, HCOs were either fixed for immunofluorescence (IF) analyses or lysed for RNA extraction and subsequently, as mentioned below, kept at −80°C for further processing.

### 2.4 In vitro SARS‐CoV‐2 replication assessment in HCO

RNA genome from SARS‐CoV‐2 infected HCO supernatants was isolated, retrotranscribed, and amplified as previously reported.^74^ Concurrently, Vero E6 cells were plated in a 96‐well plate at a density of 2 × 10^4^ cells per well. Subsequently, these cells were cultured with serial dilutions (1:3) of supernatants obtained from SARS‐CoV‐2 infected HCOs at various time points, specifically at 24‐, 48‐, and 72 hpi. After 72 h, supernatants from Vero E6 cells were collected and fixed using 4% paraformaldehyde (PFA—Sigma–Aldrich, MO, USA) for 1 h at room temperature. Cell death was then evaluated and the TCID_50_ was determined with a crystal violet solution at 0.2% (Sigma–Aldrich), as previously reported.^72^

### 2.5 iPSC‐HCO exposure to SARS‐CoV‐2 S‐protein

iPSC‐HCOs were challenged with viral S protein (0.08 μg/mL) (BEI resources). At 72 h post‐stimulation, iPSC‐HCOs were fixed for IF analyses or were lysed for RNA extraction.

### 2.6 Gene expression analyses

As reported earlier by Limanaqi et al.,^75^ total RNA was extracted and reverse transcribed from HCOs. The gene expression of 33 targets related to SARS‐CoV‐2 inflammatory, host receptors and co‐receptors, apoptotic, and antiviral pathways was assessed using Real‐time qPCR (CFX96 connect, Bio‐Rad). SYBR Green PCR mix (Promega) and specific Real‐time PCR primers (PrimePCR, Bio‐Rad, Segrate, Italy) listed in Table 2 were employed for the analysis. N1 and S1 SARS‐CoV‐2 sequences were also analyzed as well. A negative Ct value was defined as 35 or greater, and all the samples displayed GAPDH Ct values below 20; thus, none were excluded from the analysis. Gene expression analyses were assessed as previously described.^76^

**TABLE 2:** Target genes analyzed by real‐time PCR in mock, SARS‐CoV‐2 infected, and S‐exposed HCOs.

ACE2	b2‐micro	CD147	ERAP2	HLA‐A	IL10	IL37	N2	NRP1	S100B	TAP
BAX	CASP1	cGAS	FURIN	IFITM1	IL18	MAVS	NFKB	RIG1	STAT1	TOMM70
BCL2	CASP8	ERAP1	GSK3B	IFITM3	IL33	MCP1	NLRP3	S1	STAT3	VEGFA

*Note*: Data were normalized on the GAPDH housekeeping gene.

Gene expression analyses of ACE2, CD147, NRP1, and Furin were conducted using droplet digital PCR (ddPCR QX200, Bio‐Rad). This analysis can increase the detection of rare transcripts and provide absolute quantification of RNA molecules. For CD147 and NRP1 gene analyses, 3 μL of 1:100 diluted cDNA and 3 μL of ACE2 cDNA were mixed with specific primers (Qiagen, Hilden, Germany) and EvaGreen ddPCR SuperMix (Bio‐Rad). The mixture was blended with droplet generator oil (Bio‐Rad) utilizing a QX200 droplet maker, in compliance with the producer's guidelines.

The droplets were transferred to a 96‐well reaction plate, and a pierceable foil sheet (PX1, PCR plate sealer, Bio‐Rad) was utilized to heat‐seal the plate. The PCR amplification was carried out using a T100 thermal cycler (BioRad). Further steps were performed by a QX200 droplet reader (Bio‐Rad), and QuantaSoft software (version 1.7.4.0917) was used to analyze ddPCR data (Bio‐Rad). Results are shown as median and interquartile range (IQR) (25th and 75th percentile).

### 2.7 Immunofluorescence assays

Seventy‐two hours post‐infection and S‐stimulation, HCOs were fixed in PBS plus 4% PFA at 4°C overnight. One milliliter of 30% sucrose/PBS for cryopreservation was then added at 4°C overnight. The cells were then prepared for cryosection. Sections were gathered on Superfrost Plus slides. For immunostaining, the cells were washed three times in PBS and blocked for 1 h at RT with 10% normal goat serum (NGS), 0.2% Triton‐X, and 0.1% BSA in PBS. Cells were incubated overnight at 4°C with the following primary antibodies to characterize the cell model, as well as to assess SARS‐CoV‐2 host entry factors, SARS‐CoV‐2 replication, and neuronal stress conditions: TUBB3 (1:500, BioLegend), SOX2 (1:200, Abcam), PAX6 (1:100, Invitrogen), MAP2 (1:100, Merck), DCX (1:200, Abcam), Nestin (1:200, Abcam), SYNAPSIN‐1 (1:200, Merck), ACE2 (1:200, Prodotti Gianni), NRP1 (1:100, Thermo Fisher Scientific), CD147 (1:100, Thermo Fisher Scientific), CASP3 (1:200, Abcam), and SARS‐CoV‐2 nucleocapsid protein (1:1000, BEI Resources). Following, sections were stained at room temperature for 45 min with secondary antibodies (Alexa Fluor 488 or 647, 1:500, Abcam) and mounted using a medium containing DAPI (Enzo Life Sciences, Milan, Italy). Analyses by confocal microscopy were conducted using a Leica TCS SP5 AOBS microscope (Leica Microsystems, Wetzlar, Germany).

### 2.8 Statistical analyses

GraphPad Prism 8 was used to conduct the statistical analysis. The mean ± SEM of the specified n values was employed to express the results, and a threshold of 0.05 was applied when two‐tailed Student's t‐test was performed. In total, we utilized HCOs from 3 healthy subjects to perform 3 individual testing.

## 3 RESULTS

### 3.1 iPSC‐derived HCO characterization

Brain organoids represent a valuable and innovative model for studying the complexity of the human brain, closely resembling the in vivo cellular microenvironment. As a matter of fact, brain organoids can develop various discrete, although interdependent, brain regions, including the cerebral cortex.

**FIGURE 1:**
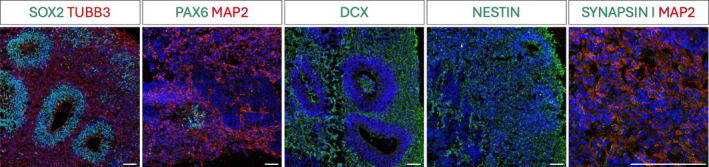
Immunofluorescence on HCOs. Immunostaining for stem cell, precursor, and neuronal markers in HCOs at day 30 (SOX2, green; TUBB3, red; PAX6, green; MAP2, red; DCX, green; NESTIN, green; Synapsin‐1, green). Nuclei were stained with DAPI, blue. Scale bar: 100 μm.

We generated iPSC‐derived HCOs from healthy controls (*n* = 3), following an established protocol.^73^ At day 30 of differentiation, iPSC‐derived HCOs were comprehensively characterized through immunocytochemistry to assess the expression of key markers related to pluripotency, neural precursor identity, and neuronal differentiation (Figure 1). Despite the process of neuronal commitment, HCOs continued to exhibit persistent expression of the pluripotency marker SOX2, specifically within progenitor cell populations. This observation highlights the heterogeneous cellular composition of the organoids, where differentiated neurons coexist with progenitor cells that retain stem cell‐like properties. Such heterogeneity is a hallmark of developing brain‐like structures, where a dynamic balance between progenitor maintenance and neuronal differentiation is crucial for proper development. The presence of SOX2 in these progenitor populations after 1 month of differentiation underlines the ongoing neurogenic potential within the organoid, reflecting the complexity and the developmental trajectory of these in vitro models which reflects the human CNS development.^53^, ^77^, ^78^ Markers associated with neuronal progenitors, such as PAX6 (paired box 6) and NESTIN, were analyzed to assess the transition to neural precursor stages, confirming the presence of a robust population of neural progenitor cells, which are crucial for the early stages of brain development. These markers indicate that the organoids successfully recapitulate the neurogenic niches typically observed during the embryonic development of the human brain.

Additionally, the detection of neuronal differentiation markers, including TUBB3 (beta‐III tubulin), MAP2 (microtubule‐associated protein 2), DCX (doublecortin), and Synapsin‐1, provide evidence of advanced neuronal development. TUBB3 and MAP2 expression revealed the formation of differentiated neurons with stabilized microtubule networks, essential for axonal and dendritic growth. The presence of DCX, a marker of migrating neurons, suggests that neuronal migration is occurring within the organoids. Additionally, Synapsin‐1 expression is indicative of synaptogenesis, pointing to the establishment of functional connections between neurons and the emergence of nascent neuronal networks.

These findings collectively demonstrate that the organoids not only exhibit a heterogeneous cellular composition, encompassing both progenitor and mature neuronal populations but also mirror key developmental processes such as neurogenesis, neuronal migration, and synaptogenesis.

### 3.2 SARS‐CoV‐2‐specific human receptor and co‐receptor expression in HCOs

**FIGURE 2:**
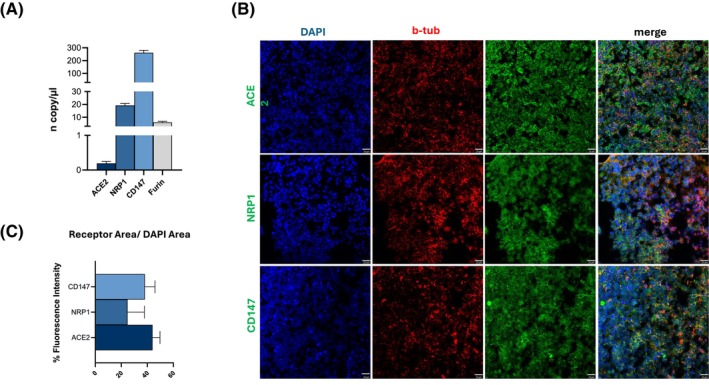
Expression of human SARS‐CoV‐2 primary entry factors on HCOs. (A) Digital gene expression analyses were conducted on iPSC‐HCOs to investigate the mRNA expression of ACE2, NRP1, CD147, and Furin entry factors targeted by SARS‐CoV‐2. Results are shown as copy/ul. and derived from a minimum of three separate experimental runs each one on HCOs obtained from 3 healthy individuals. (B) Representative IF images of ACE2, CD147, and NRP1 markers (green) and nuclei in DAPI (blue) in HCOs. Bars are equivalent to 20 μm. (C) Quantification of ACE2, CD147, and NRP1 receptor fluorescence intensity, expressed in percentage, obtained by analyzing the ratio of receptor area/DAPI area from three different images acquired from the same HCO section.

The assessment of the expression of key human receptors, co‐receptors (ACE2, CD147, and NRP1), and the peptidase (Furin) employed by SARS‐CoV‐2^79^, ^80^ was performed on HCOs through digital PCR. HCOs expressed each of these entry factors but with different gene expression levels (Figure 2A). Specifically, ACE2 and Furin were substantially less expressed than CD147 and NRP1, according to the gene expression study. However, protein expression of the SARS‐CoV‐2 entry factors assessed by IF assay (Figure 2B) did not confirm this finding. Indeed, ACE2 expression was slightly higher compared to CD147 and NRP1 (Figure 2C), suggesting the occurrence of compensatory cellular mechanisms balancing mRNA transcription and protein translation turnover.

### 3.3 SARS‐CoV‐2 Infection and Replication in HCOs

**FIGURE 3:**
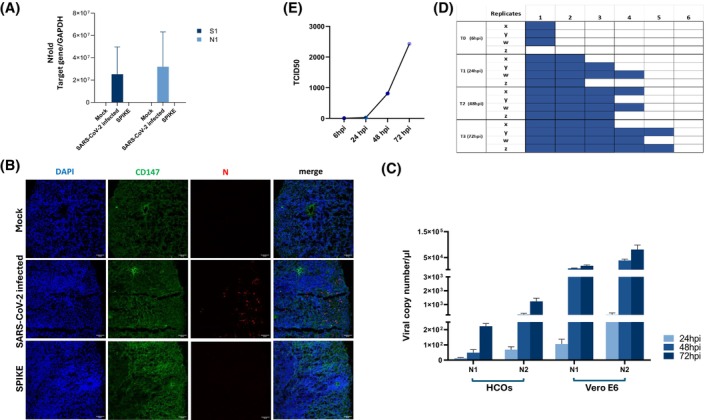
Assessment of SARS‐CoV‐2 in vitro infection and replication in HCOs. (A) mRNA expression of S1 (Spike) and nucleocapsid (N1) viral sequences were investigated in SARS‐CoV‐2‐infected, spike (S)‐exposed, and uninfected (Mock) HCOs by real‐time qPCR. Results are displayed as the average value plus standard error of the mean (SEM) derived from a minimum of three separate experimental runs each one on HCOs obtained from 4 healthy individuals. (B) Comparative immunofluorescence imaging of nucleocapsid viral protein (N) (red), CD147 receptor (green), and nuclei (blue) in uninfected (Mock), infected with SARS‐CoV‐2, and S‐exposed HCOs 72‐hpi. Bars correspond to 60 μm. (C) Viral replication was evaluated at 24‐, 48‐ and 72‐h post‐infection (hpi) in SARS‐CoV‐2‐infected HCO and Vero E6 supernatants (MOI 0.001). Viral copies per microliter were assessed by real‐time PCR quantifying two regions of the SARS‐CoV‐2 nucleocapsid gene (N1 and N2). Data are displayed as the average value plus standard error of the mean (SEM) and were derived from a minimum of four separate experimental runs. (D) TCID_50_ analyses were conducted to assess the infectivity of VeroE6 cells following exposure to SARS‐CoV‐2‐infected HCO‐supernatants. This included the undiluted supernatant (1) and 5 serial 1:3 dilutions, at 6, 24, 48, and 72 hpi. The presented plate serves as a representation of a singular experiment performed in quadruplicate (X, Y, W, Z). Analyses of the cytopathic effect, represented by colored well in blue, were used to verify Vero E6 infectability by SARS‐CoV‐2. (E) The quantification of SARS‐CoV‐2 in the Vero E6 supernatants was performed at 6, 24, 48, and 72 hpi, using the data reported in (D). TCID_50_ values are displayed, with results reported as the average value with the standard error of the mean (SEM) and were derived from four separate experimental runs each one on HCOs obtained from four healthy individuals.

Different experimental approaches were employed to verify if HCOs could be successfully infected by SARS‐CoV‐2. First, the presence of specific viral products within HCOs was evaluated at the intracellular level using real‐time qPCR and IF approaches. The first one confirmed mRNA expression of SARS‐CoV‐2 nucleocapsid (N1) and Spike (S1) target sequences in infected HCOs (Figure 3A). Likewise, the viral nucleocapsid (N) protein was identified solely in SARS‐CoV‐2 infected HCOs, predominantly localized in the external region (Figure 3B and Supplementary Figure 1), though the percentage of infected cells was apparently scant.

In parallel, gene expression analyses of both N1 and N2 viral nucleocapsid sequences in supernatants from HCO cultures, over a 72‐hpi period, proved an effective and progressive SARS‐CoV‐2 infection and replication in HCOs (Figure 3C).

**FIGURE 4:**
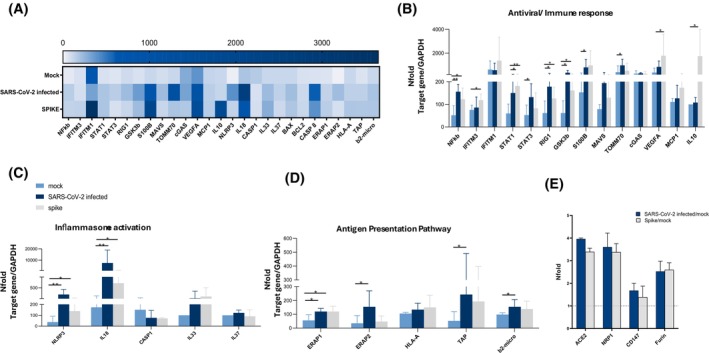
Transcriptional pathways altered in HCOs post SARS‐CoV‐2‐infection or S‐exposure. Gene expression analyses. (A) At 72 h post‐infection, mRNA levels of 27 different genes were evaluated in HCOs uninfected (Mock), infected with SARS‐CoV‐2 and stimulated with the Spike protein. Results are displayed in a Heatmap. Gene expression (nfold) is shown as a color scale from white to dark blue (0 to +3500). Real‐time qPCR was employed to quantify mRNA levels of genes related to the immune and antiviral response (B), inflammasome activation (C), antigen processing and presentation pathway (D). As for entry factors NFold of ACE2, NRP1, CD147, and Furin peptidase, measured by real‐time‐PCR, was reported as an increase of the HCOs SARS‐CoV‐2 infected, and S‐exposed conditions in comparison to the uninfected one (E). The 2^−ΔΔCt^ equation was used to quantify expression level. Data are displayed as the average value with the standard error of the mean (SEM) and were derived from a minimum of three separate experimental runs each one on HCOs obtained from 4 healthy individuals. *p < 0.05; **p < 0.01.

Nevertheless at 72 hpi, SARS‐CoV‐2 replication in HCOs was significantly lower (average SARS‐CoV‐2 copy number/ul ± SEM: N1 = 222.99 ± 17.64; N2 = 1229.38 ± 228.17) than that detected in susceptible Vero E6 cells (average SARS‐CoV‐2 copy number/ul ± SEM: N1 = 15.17e+06 ± 2.2 e+06; N2 = 77.94e+06 ± 19.27 e+06) (Figure 4).

To further confirm HCO's potential to sustain the productive infection, supernatants gathered from infected HCOs at 6, 24, 48, and 72 hpi were used to re‐infect Vero E6 cells. As a result, Vero E6 cells showed a clearly visible cytopathic effect, which increased throughout the supernatant collecting period (Figure 3D). Indeed, the virus titers displayed a rise from 27 TCID_50_ at 24 hpi to 2430 TCID_50_ at 72 hpi (Figure 3E), replicating the outcomes of real‐time qPCR.

### 3.4 Transcriptome analyses in HCO following in vitro SARS‐CoV‐2‐infection or S‐exposure

To assess whether SARS‐CoV‐2 infection or S‐exposure alters cellular homeostasis and metabolism, including the antiviral and immune response in HCOs, we evaluated the expression of 31 different genes of interest at 72 h post‐infection or stimulation. Results summarized in Figure 4A demonstrate that both SARS‐CoV‐2 infection and S‐stimulation foster transcriptional dysregulation in several of the analyzed targets. Notably, the trend of the various targets exhibited a similar pattern following both SARS‐CoV‐2 infection and S‐exposure (Figure 4A).

In detail, results show that both SARS‐CoV‐2 infection and S‐exposure augmented the mRNA expression of many intracellular targets peculiar to virus contact, including interferon‐induced transmembrane proteins (IFITM3 and IFITM1), interferon‐stimulated genes (NFKB, STAT1, STAT3, and RIG1), as well as a neuronal stress‐related marker (S100B), and GSK3b, a multifunctional kinase involved in neuronal homeostasis, differentiation, and metabolism (Figure 4B). We also observed a consistent rise in NLRP3 and IL‐18 mRNA expression, which are key components of the inflammasome pathway (Figure 4C).

Of note, significant modulation of genes implicated in the antigen processing and presentation pathway (ERAP1, ERAP2, TAP, and b2‐microglobulin) was observed as well (Figure 4D). Interestingly, the induction of this pathway was evident following both viral infection and S exposure, but statistically significant differences were evident post‐SARS‐CoV‐2 infection alone. This implies an active viral replication is needed to fully boost antigen processing and presentation.

Moreover, as shown in Figure 4E, following 72 h of either SARS‐CoV‐2 infection or S‐stimulation, we observed, by real‐time PCR, a generalized increase in the expression of all human SARS‐CoV‐2 entry factors compared to the mock HCOs.

### 3.5 Apoptotic pathway alteration in SARS‐CoV‐2‐infected or S‐exposed HCOs

**FIGURE 5:**
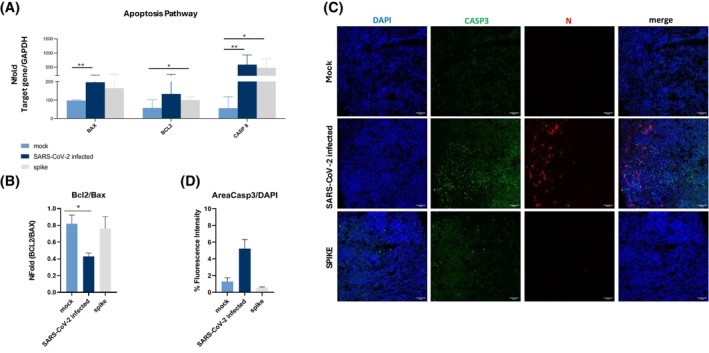
Apoptotic pathway alteration in SARS‐CoV‐2‐infected or S‐exposed HCOs. (A) Real‐time qPCR was utilized to quantify mRNA levels of genes related to the apoptotic pathway (BAX, BCL2 CASP8), in uninfected (Mock), SARS‐CoV‐2‐infected, and S‐exposed HCOs. The 2^−ΔΔCt^ equation was used to quantify expression level. (B) The ratio between mRNA from the anti‐apoptotic (BCL2) and pro‐apoptotic (BAX) genes was analyzed at 72 hpi or spike stimulation. Data are displayed as the average value with the standard error of the mean (SEM) and were derived from a minimum of three separate experimental runs. *p < 0.05; **p < 0.01. (C) Caspase 3 protein (green), viral nucleocapsid protein (red), and nuclei (blue) analyzed by immunofluorescence assay in Mock, SARS‐CoV‐2‐infected, and S‐exposed HCOs at 72 hpi. Bars are equivalent to 60 μm. (D) Quantification of Caspase 3 fluorescence intensity, expressed in percentage, obtained by analyzing the ratio of Casp3 area/DAPI area from three different images acquired from the same HCO section.

To further determine the effects of viral infection and S‐exposure on HCO viability, we investigated the apoptotic pathway 72 h post‐infection or stimulation. Thus, the mRNA expression of some pro‐apoptotic genes, including BAX, BCL2, and Caspase 8, was established. Results showed that all these genes were upregulated upon both SARS‐CoV‐2 infection and S‐protein exposure (Figure 5A). A significant decrease in the BCL2/BAX ratio was nevertheless observed only after HCO SARS‐CoV‐2 infection, suggesting various plausible speculations about how viral entry affects the apoptotic pathway (Figure 5B).

Through IF analyses, we assessed the expression of Caspase 3, a key modulator of apoptosis in neuronal cells and organoids. As demonstrated by the representative IF panel (Figure 5C) and corroborated by fluorescence quantification analysis (Figure 5D), Caspase 3 expression was significantly increased in HCOs following SARS‐CoV‐2 infection compared to the uninfected (mock) condition.

## 4 DISCUSSION

While the blood–brain‐barrier (BBB) serves as a highly effective safeguard, preventing peripheral immune cells and external molecules from entering the brain parenchyma, some viruses, including the highly pathogenic coronaviruses MERS and SARS, have developed neuroinvasive abilities to bypass this protective barrier and infect the CNS. This, in turn, causes both immediate and long‐term adverse outcomes.^81^, ^82^, ^83^, ^84^ Likewise, neurological symptoms, like dementia or encephalopathy, reported during the recent SARS‐CoV‐2 pandemic, suggest that this coronavirus could injure the central as well as the peripheral nervous systems. It is hypothesized that an excessive inflammatory response plays a major role in this context; however, direct infection of neural cells may also contribute to the onset of neurological symptoms. Notwithstanding, there is limited and conflicting information regarding the impact of SARS‐CoV‐2 infection on neuronal homeostasis. For example, Pedrosa et al. reported that SARS‐CoV‐2 has a constrained capacity for neuronal infection, while astrocyte infectability was evident and triggered inflammatory responses and neuronal damage.^85^ Conversely, in several in vitro cell culture models, SARS‐CoV‐2 was proved capable of efficiently infecting neurons derived from both human embryonic stem cells and iPSCs.^62^, ^71^, ^86^, ^87^ To further dissect this controversial issue, we take advantage of human iPSC‐derived brain organoids, which are in vitro 3D models mimicking the molecular, cellular, and functional aspects of the human brain.

We first assessed the presence of different entry factors (ACE2, NRP1, CD147, and Furin) exploited by SARS‐CoV‐2 to infect cells on HCOs. Gene expression profiling and IF techniques showed that all these molecules are expressed on HCOs. Moreover, their expression was consistently induced following both SARS‐CoV‐2 infection and S‐exposure, indicating that HCOs recognize the virus/viral components and react to such encounters. Though preliminary, these findings suggest that SARS‐CoV‐2 may directly infect HCOs, potentially exploiting the interactions with each of the entry factors analyzed. This result is particularly relevant considering that CD147 and NRP1 proteins are significantly expressed in the human brain,^88^ and their utilization by SARS‐CoV‐2 has been extensively documented.^89^, ^90^, ^91^, ^92^, ^93^ Newly emerging evidence, indeed, indicates that CD147 and NRP1 proteins display a more comprehensive and widespread expression pattern in the human brain compared to ACE2.^88^, ^89^, ^90^, ^94^, ^95^, ^96^, ^97^, ^98^, ^99^

In line with this speculation, herein we demonstrated through different experimental approaches that SARS‐CoV‐2 productively infects HCOs and replicates in this 3D cellular model. These findings corroborate the previously established evidence of productive SARS‐CoV‐2 infection and replication reported in the 2D neuronal cortical HCN‐2 cell line,^100^ as well as in HCO conducted by other research groups.^52^, ^59^, ^61^, ^67^, ^68^, ^71^, ^101^ The viral replication rate in HCOs was notably constrained compared to that observed in cell lines known for their susceptibility to SARS‐CoV‐2 infection, such as Vero E6 cells. However, in this 3D model, the immunological components, mainly represented by astrocytes and microglia, are missing. Microglia, the primary immune cells in the brain, swiftly activate in response to viral infections, creating a pro‐inflammatory milieu where cytokines, chemokines, and reactive oxygen species potentially enhance viral infection and replication, leading to neuronal damage.^37^, ^40^, ^49^, ^50^, ^102^, ^103^, ^104^, ^105^, ^106^, ^107^, ^108^ Therefore, it is conceivable that under pro‐inflammatory conditions, particularly in individuals with pre‐existing neuronal pathology, there might be a substantial escalation in viral entry and replication mechanisms.

At the same time, it should be underlined that in this HCO model, the protective effect physiologically exerted by the BBB is completely absent. Previous findings have consistently demonstrated SARS‐CoV‐2's capacity to pass through the BBB using a transcellular pathway, leading to an amplification of the inflammatory response.^49^, ^62^, ^66^, ^109^, ^110^, ^111^, ^112^ Analyses performed in cerebrospinal fluid (CSF) of COVID‐19 patients, nevertheless, revealed the presence of viral particles exclusively in individuals with concurrent pathologies linked to BBB impairment, emphasizing the crucial role of the BBB in preventing SARS‐CoV‐2 infection.^113^ To mimic the few viral particles, that are expected to cross the BBB, we performed the in vitro HCO‐SARS‐CoV‐2 infection assay with a very low viral input (0.001 MOI). However, further experiments in more complex human brain organoid models, designed to recapitulate the vasculature and microenvironment of the BBB, are currently underway and are expected to provide further insights into the ability of SARS‐CoV‐2 to breach the BBB and infect neurons.

In our model, we observed that iPSC‐HCO homeostasis is altered even with very low viral input. In fact, transcriptomic analyses showed a relevant modification in the expression of genes partaking in different intracellular pathways. Among the upregulated genes some are involved in signaling transduction (NFKB, STAT1, and STAT3) and interferon pathway (IFITM1 and IFITM3) and are recognized as essential components of protective antiviral responses.^114^, ^115^ As these pathways are triggered even just following S‐exposure, these results suggest that HCOs can recognize viral particles and initiate defensive responses even in the lack of a productive infection.

Notably, both infection and S‐exposure also upregulated the expression of the neuronal stress marker S100B. This calcium‐binding protein is released in biological fluids and serves as a consistent indicator of stressful conditions. Indeed, increased S100B serum concentrations have been associated with COVID‐19 severity and neuronal damage.^59^, ^116^, ^117^ The initiation of an inflammatory reaction in SARS‐CoV‐2‐infected HCOs was further substantiated by the increased expression of genes associated with the inflammasome pathway, particularly NLRP3 and IL‐18. Notably, NLRP3 inflammasome activation has been observed in the brains of SARS‐CoV‐2‐infected patients,^37^, ^118^, ^119^ signifying its pivotal involvement in CNS lesions associated with SARS‐CoV‐2. Of note, as the expression of these markers was increased even following S‐exposure, it is worthwhile to wonder if, even in the absence of overt infection, Spike alone could induce neurotoxic effects in the CNS, as already observed in other cellular models.^120^, ^121^, ^122^, ^123^, ^124^, ^125^, ^126^ Indeed, it has already been demonstrated that the S protein could alter the barrier function,^35^ as well as cross the BBB and enter the brain in mice.^127^

The antigen presentation pathway also exhibited significant activation following SARS‐CoV‐2 infection, as evidenced by the increased ERAP1/ ERAP2, TAP, and β2‐microglobulin expression. This result is not unexpected, as the modulation of HLA‐I and ERAP expression has already been documented in several cell lines following viral infections, including SARS‐CoV‐2,^76^, ^128^ and is generally considered as a protective mechanism activating viral‐specific lymphocyte responses. However, in the CNS, this phenomenon could display negative repercussions. Neuronal HLA‐I expression plays a central role in the formation, function, and alteration of synapses; yet, an abnormal increase is known to occur upon immune activation, including viral infections, and also during neurodegeneration.^129^ HLA‐I presents viral antigens to CD8+ T lymphocytes and activates cytotoxicity to prevent lethal viral spread.^130^ Excessive or misdirected cytotoxicity can nevertheless be responsible for autoimmune responses, potentially supporting a role for virus‐induced HLA‐I upregulation in the development of neurodegenerative diseases.^129^, ^131^ Therefore, in the future, it will be interesting to verify whether HLA‐I upregulation contributes to SARS‐CoV‐2‐associated neuropathology.

Finally, the disruptions of neuronal homeostasis caused by either HCO SARS‐CoV‐2 infection or S‐protein exposure also promoted the upregulation of Caspase 8, BAX, and BCL2 expression, mediators of the apoptotic pathway. It is noteworthy, though, that the anti‐apoptotic (BCL2)/pro‐apoptotic (BAX) ratio significantly decreased only in SARS‐CoV‐2‐infected HCOs, indicating that after infection, apoptosis is somewhat enhanced in this neural model. These results seem to confirm previous findings showing that SARS‐CoV‐2‐infected neurons develop cellular stress, promoting cell death.^59^, ^62^, ^64^, ^68^, ^132^ Likewise, Li et al. observed an increase in Caspase 8 levels, responsible for apoptosis and inflammation, in SARS‐CoV‐2‐infected lung epithelial cells, indicating a cellular response to viral infection.^133^ Remarkably, in our 3D model, we also observed a significant rise in the expression of Caspase 3, a crucial mediator of apoptosis in neuronal cells and organoids.^59^, ^101^, ^112^ Considering its substantial role in the break of amyloid‐beta A precursor, which is linked to Alzheimer's disease‐related neuronal degeneration,^134^, ^135^ the augmented expression of this protein after HCO SARS‐CoV‐2 infection could potentially be correlated with the initiation of subsequent neurological symptoms.

Overall, these findings confirm that HCOs may be infected by SARS‐CoV‐2, further supporting other studies in the fields that also showed a significant infection of cells such as cortical neurons, astrocytes, and certain neuronal progenitor population,^59^, ^65^, ^68^, ^70^, ^71^, ^98^, ^101^, ^112^, ^132^, ^136^, ^137^, ^138^ mainly localized in the periphery^139^ of brain organoids. Such infection potentially occurs by exploiting the interaction with ACE2, CD147, and NRP1 proteins. Furthermore, exposure to the S protein alone exhibited the capacity to disrupt their equilibrium and induce neurotoxic effects, potentially playing a role in the onset of long‐COVID symptoms.

However, SARS‐CoV‐2 has a wide range of viral proteins, beyond the spike, that can induce host immune responses even without direct infection. For instance, current researches indicate that nucleocapsid protein plays a significant role in modulating immune responses.^140^, ^141^, ^142^, ^143^ It would, therefore, be interesting to test the effects of other viral proteins, primarily the N protein on HCOs, to further explore the “armamentarium” of SARS‐CoV‐2 and its effects on the host immune system. Moreover, it is worthwhile to consider that the disrupted phenotype caused by SARS‐CoV‐2 infection/replication could be massively diluted by most of the cells which, according to IF data, are not infected. In the future, we, therefore, intend to verify how viral infection/replication affects mRNA transcription, by adopting a single‐cell approach.

Despite the acknowledged limitations, this study offers relevant insights into the molecular mechanisms that might be behind the neurological symptoms of COVID‐19 and long‐COVID, providing an opportunity to identify novel therapeutic targets to prevent or control the onset of neurological symptoms.

## AUTHOR CONTRIBUTIONS

Mara Biasin and Gioia Cappelletti conceived and designed the research; Gioia Cappelletti, Lorenzo Brambilla, and Sergio Strizzi performed the research and acquired the data; Gioia Cappelletti, Lorenzo Brambilla, and Sergio Strizzi with the help of Valentina Melzi, Mafalda Rizzuti, Monica Nizzardo, Irma Saulle, and Fiona Limanaqi analyzed and interpreted the data. Gioia Cappelletti wrote the manuscript; Mara Biasin, Stefania Corti, Daria Trabattoni, Lorenzo Brambilla, Valentina Melzi, and Mafalda Rizzuti were involved in drafting and revising the manuscript. Mara Biasin and Stefania Corti supervised the project. Mario Clerici contributed to the funding acquisition. All authors discussed the results and contributed to the final manuscript.

## FUNDING INFORMATION

Partially supported by Dino Ferrari Center; PNRR‐Spoke 13‐CUP‐G43C2200260007‐INF‐ACT.

## DISCLOSURES

The authors have no relevant financial or non‐financial interests to disclose.

## ETHICS STATEMENT

Ethical clearance was obtained from the University of Milan Ethics Committee (number 14/22). Written informed consent was obtained after receiving information about the use of their biological samples. The biological material was anonymized.

## CONSENT FOR PUBLICATION

All authors give consent for the publication of the manuscript.

## Supporting information


Data S1.

## Data Availability

The datasets used and/or analyzed during the current study are available from the corresponding author on reasonable request.
