# Supplementary material for: iPSC‐derived human cortical organoids display profound alterations of cellular homeostasis following SARS‐CoV‐2 infection and Spike protein exposure
Source: FASEB J. 2025 Feb 14;39(4):e70396. doi: 10.1096/fj.202401604RRR (PMC11826378; doi:10.1096/fj.202401604RRR)
Supplement: Supplementary file 1 — Data S1. [file FSB2-39-e70396-s001.pdf]

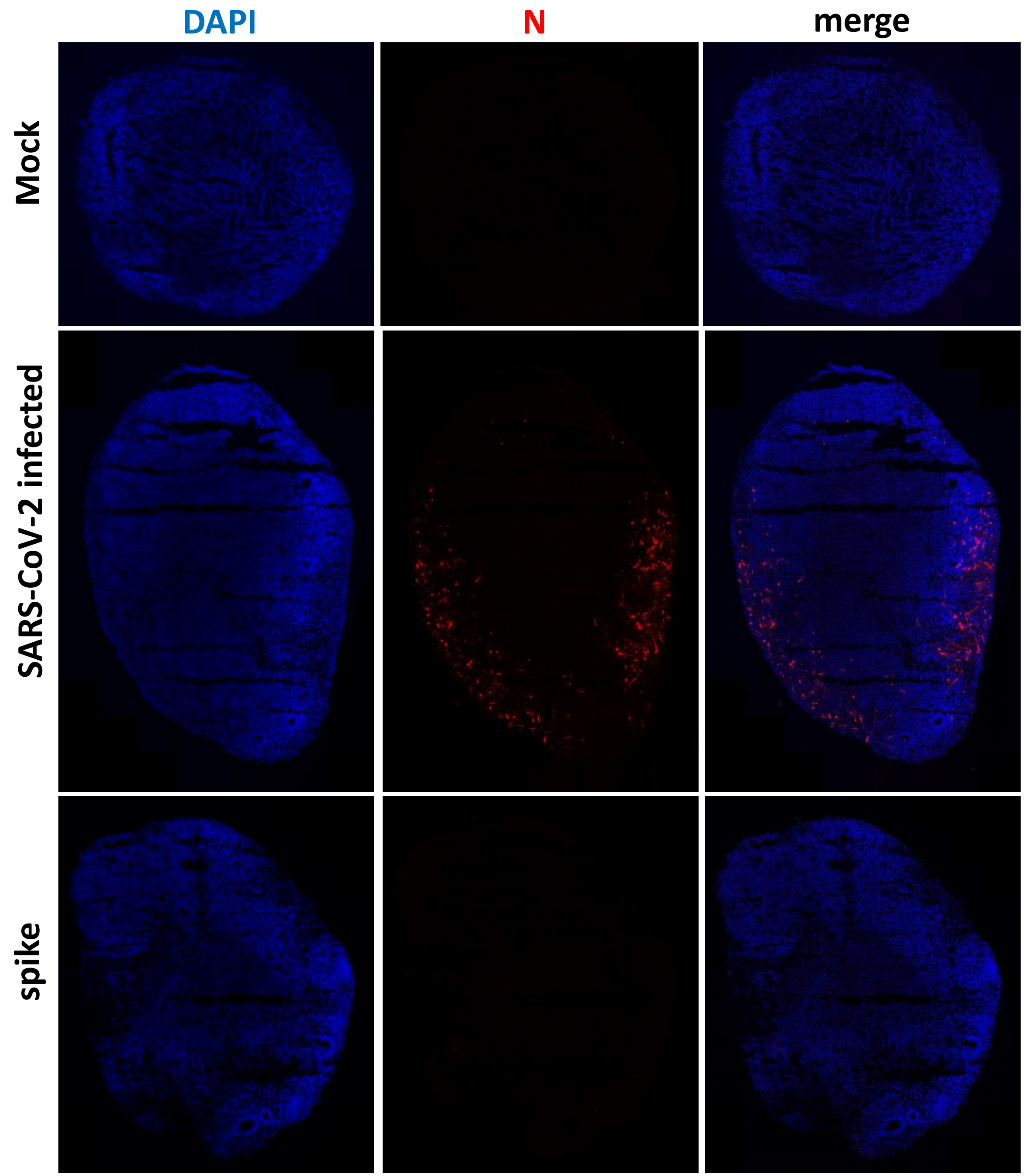

Supplementary Fig. 1 . *In vitro* SARS-CoV-2 infection in iPSC-Human cortical organoids. Intracellular analysis. Representative immunofluorescence images of N protein (red) in Mock, SARS-CoV-2-infected and S-exposed iPSC-HCOs at 72 hpi. Nuclei were stained with DAPI (blue).
